# Sum of boxes of the clinical dementia rating scale highly predicts conversion or reversion in predementia stages

**DOI:** 10.3389/fnagi.2022.1021792

**Published:** 2022-09-23

**Authors:** Ray-Chang Tzeng, Yu-Wan Yang, Kai-Cheng Hsu, Hsin-Te Chang, Pai-Yi Chiu

**Affiliations:** ^1^Department of Neurology, Tainan Municipal Hospital, Tainan, Taiwan; ^2^Department of Neurology, China Medical University Hospital, Taichung, Taiwan; ^3^Department of Medicine, China Medical University, Taichung, Taiwan; ^4^Artificial Intelligence Center for Medical Diagnosis, China Medical University Hospital, Taichung, Taiwan; ^5^Department of Psychology, College of Science, Chung Yuan Christian University, Taoyuan City, Taiwan; ^6^Department of Neurology, Show Chwan Memorial Hospital, Changhua, Taiwan; ^7^Department of Applied Mathematics, Tunghai University, Taichung, Taiwan

**Keywords:** the clinical dementia rating, sum of boxes of the clinical dementia rating, Alzheimer’s disease, history-based artificial intelligence clinical dementia diagnostic system, predementia

## Abstract

**Background:**

The clinical dementia rating (CDR) scale is commonly used to diagnose dementia due to Alzheimer’s disease (AD). The sum of boxes of the CDR (CDR-SB) has recently been emphasized and applied to interventional trials for tracing the progression of cognitive impairment (CI) in the early stages of AD. We aimed to study the influence of baseline CDR-SB on disease progression to dementia or reversion to normal cognition (NC).

**Materials and methods:**

The baseline CDR < 1 cohort registered from September 2015 to August 2020 with longitudinal follow-up in the History-based Artificial Intelligence Clinical Dementia Diagnostic System (HAICDDS) database was retrospectively analyzed for the rates of conversion to CDR ≥ 1. A Cox regression model was applied to study the influence of CDR-SB levels on progression, adjusting for age, education, sex, neuropsychological tests, neuropsychiatric symptoms, parkinsonism, and multiple vascular risk factors.

**Results:**

A total of 1,827 participants were analyzed, including 1,258 (68.9%) non-converters, and 569 (31.1%) converters with mean follow-up of 2.1 (range 0.4–5.5) and 1.8 (range 0.3–5.0) years, respectively. Conversion rates increased with increasing CDR-SB scores. Compared to a CDR-SB score of 0, the hazard ratios (HR) for conversion to dementia were 1.51, 1.91, 2.58, 2.13, 3.46, 3.85, 3.19, 5.12, and 5.22 for CDR-SB scores of 0.5, 1.0, 1.5, 2.0, 2.5, 3.0, 3.5, 4.0, and ≥4.5, respectively (all *p* < 0.05 except for CDR-SB score = 0.5). In addition, older age, lower education, lower cognitive performance, and a history of diabetes also increased conversion rates. Furthermore, reversions to NC were 12.5, 5.6, 0.9, and 0% for CDR-SB scores of 0.5, 1.0–2.0, 2.5–3.5 and ≥4.0, respectively (*p* < 0.001).

**Conclusion:**

CDR-SB in predementia or very mild dementia (VMD) stages highly predicts progression to dementia or reversion to NC. Therefore, CDR-SB could be a good candidate for tracing the effectiveness of pharmacological and non-pharmacological interventions in populations without dementia.

## Introduction

Prediction or prevention factors for progression to dementia in older adults has attracted attention of most clinicians and researchers in this field (Uhlmann et al., 1989; Aggarwal et al., 2006; Potter and Steffens, 2007; Vesely and Rektor, 2016). Good predictors help prevent disease progression; therefore, prevention studies using either pharmacological or non-pharmacological interventions have committed to develop or identify the most sensitive tools that can help trace the effectiveness of the interventions more accurately (Cedarbaum et al., 2013; Banks et al., 2020; Lu et al., 2021). Candidate factors, including clinical information (Uhlmann et al., 1989; Aggarwal et al., 2006; Potter and Steffens, 2007; Vesely and Rektor, 2016), liquid biomarkers (Wolfsgruber et al., 2017; Caminiti et al., 2018; Tible et al., 2020; Cullen et al., 2021), and imaging biomarkers (Iaccarino et al., 2017; Sanchez-Catasus et al., 2017; Caminiti et al., 2018; de la Torre, 2018), have been widely studied in the past few decades. Among these predictors, clinical information, including data on neuropsychological performance and detailed personal history acquired from participants themselves or their informants, is direct and cost-effective and can be widely used in clinical settings or for research purposes (Dickerson et al., 2007; Hung et al., 2021).

The clinical dementia rating (CDR) scale is a commonly used diagnostic tool for staging dementia due to Alzheimer’s disease (AD) (Morris, 1993, 1997; Morris et al., 1997; O’Bryant et al., 2008, 2010). During assessment, six cognitive or functional domains, including memory, orientation, judgment, community affairs, home hobbies, and personal care, are scored by trained physicians after interviewing both participants and their informants. Thus, function- and performance-based information is acquired simultaneously. Subjective cognitive impairment (CI) and objective cognitive deterioration, which are essential for the diagnosis of mild cognitive impairment (MCI) or dementia, can be confirmed after assessment (Albert et al., 2011; McKhann et al., 2011). However, owing to the lack of frontal behavior or language domain, it is less applied or occasionally modified to stage CI or dementia due to non-AD disorders (Maiovis et al., 2017; Mioshi et al., 2017).

The sum of boxes of the CDR (CDR-SB) is the sum score of the six domains. The clinical values of the CDR-SB for diagnosis or tracing CI/dementia progression are still being investigated (O’Bryant et al., 2008, 2010; Cedarbaum et al., 2013; Banks et al., 2020; Lu et al., 2021; Yang et al., 2021). For the diagnosis of different CI stages including MCI and dementia, O’Bryant et al. (2010) first used the CDR-SB and determined the cutoff scores for normal cognition (NC) versus MCI and MCI versus dementia among individuals with AD as well as non-AD. They concluded that the CDR-SB has a fair diagnostic power in another multicenter trial (Albert et al., 2011). Recently, our group has also investigated the diagnostic value of the CDR-SB for differentiating CI stages from subjective cognitive decline (SCD) to MCI and subsequently dementia among participants with AD and without AD; we found fair and similar diagnostic power for determination of CI stages among individuals without AD compared to that among individuals with AD (Yang et al., 2021). In addition, in some clinical trials tracing the conversion of people in predementia stages, the CDR-SB has been suggested as a primary endpoint for the tracing of effectiveness in these studies (Cedarbaum et al., 2013; Banks et al., 2020; Lu et al., 2021).

In the current study, based on the recent evidence of good diagnostic power for discrimination of different CI stages due to AD and non-AD, we initially investigated the contribution of different CDR-SB levels in individuals without dementia using a longitudinal follow-up study. Subsequently, we determined the reliability of different CI stages using the CDR-SB by tracing the rates of conversion to dementia or reversion to NC. Furthermore, we expected to identify the point of no return to NC determined by the CDR-SB.

## Materials and methods

This study based on the data from the History-based Artificial Intelligence Clinical Dementia Diagnostic System (HAICDDS) project was retrospective with longitudinal follow-up. HAICDDS is currently used for the registration of patients with dementia in four hospitals of the Show Chwan Healthcare System in Taiwan (Chiu et al., 2019; Zhu et al., 2020; Yang et al., 2021). Purpose of the project was to register the participants with CI in a structured and standardized form for further machine learning or deep learning to improve the diagnosis of stages and subtypes of dementia. Therefore, normal people and the patients with CI due to AD, cerebrovascular disease (CVD), Lewy body disease (LBD), or other brain disorders were consecutively registered. All participants and their informants were interviewed by neuropsychologists with well training and requested to complete a neuropsychological tests and surveys of activities of daily living. Apart from the CDR scale (Morris, 1993), neuropsychological evaluations including the Cognitive Abilities Screening Instrument (CASI) (Lin et al., 2002), History-based Artificial Intelligence Activities of Daily Living (HAIADL) (Hung et al., 2021), Montreal Cognitive Assessment (MoCA) (Nasreddine et al., 2005), Instrumental Activities of Daily Living (IADL) scale (Lawton and Brody, 1969), and Neuropsychiatric Inventory (NPI) (Cummings, 1997) were applied to assess the severity of CI or dementia. In this study, we will select and analyze a cohort without dementia with longitudinal follow-up data.

### Diagnosis of normal cognition, subjective cognitive decline, mild cognitive impairment, and dementia in the history-based artificial intelligence clinical dementia diagnostic system database

Normal cognition is diagnosed as having a global CDR (Morris, 1993) score of 0. The CASI should be in the non-demented range after adjustment for age, sex, and education (Lin et al., 2002). MCI was diagnosed based on the criteria proposed by Petersen et al. (1999). It is defined as the symptomatic predementia phase of AD, thus there is a change in cognition with impairment in the CASI or MoCA tests, but without impairment in social or occupational functioning with a CDR score of 0.5 (Albert et al., 2011). The cutoff scores of MCI using the CASI after adjustment for age and education should be in the non-demented range (Lin et al., 2002). Dementia was diagnosed based on the criteria proposed by the NIA-AA (McKhann et al., 2011). Participants were considered to have dementia when having the impairments in two or more cognitive domains and a decline in daily functions with a global CDR score ≥ 0.5. IADL score < 6 or HAIADL score > 8 were applied for the operational diagnosis of functional impairment (Lawton and Brody, 1969; Morris et al., 1997). The CASI was used to define CI. The cutoff score after adjustment for age, sex, and education should be in the demented range (Lin et al., 2002).

### Definition of conversion and reversion

The first assessment for both converters and non-converters is the baseline. Assessment in each follow-up is a checkpoint. The conversion is defined by a complete assessment conforming with the cutoff scores of CDR ≥ 1 for dementia. After adjusting for age and education, the CASI score should be lower than the cutoff scores (Lin et al., 2002). In addition, the scores of CDR-SB, CASI, MoCA, and IADL should be worse at the later checkpoints than those at baseline. The endpoint for non-converters is defined as the final checkpoint. The endpoint for converters is defined as the checkpoint that fulfils the criteria for conversion without return to the non-dementia stages. Reversion is defined as any follow-up assessment that reverses to CDR = 0.

### Study procedure and determination of normal cognition, subjective cognitive decline, mild cognitive impairment, very mild dementia, and dementia using the sum of boxes of the clinical dementia rating

**FIGURE 1:**
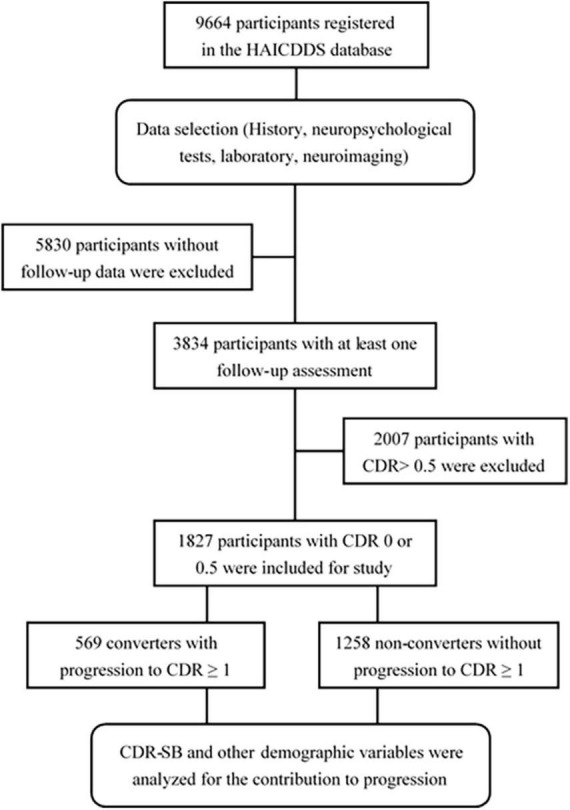
Flow chart for participant selection.

The clinical dementia rating < 1 cohort registered from September 2015 to August 2020 with at least one follow-up assessment was collected and investigated. The following data were analyzed: (1) demographic data including sex, age, education, follow-up period, and history of other relevant diseases, such as parkinsonism, CVD, hypertension, carotid artery disease, congestive heart failure, arrhythmia, diabetes, and dyslipidemia and (2) the results of CDR-SB and neuropsychological tests including CASI, IADL, NPI, and MoCA. Converters and non-converters were identified, and the hazard ratios (HRs) for all scores of the CDR-SB in global CDR = 0 or 0.5 and demographic variables were analyzed. CI stages grouping with the CDR-SB were determined according to the previous studies by O’Bryant et al. (2008, 2010) and Yang et al. (2021). CDR-SB levels of 0, 0.5, 1.0–2.5, 2.5–4.0, and ≥4.5 were defined as NC, SCD, MCI, very mild dementia (VMD), and dementia, respectively. Conversion and reversion rates among different CI stages grouped using the CDR-SB were also derived and compared. The detailed procedure is shown in Figure 1.

### Statistics

The Chinese version of IBM SPSS Statistics for Windows, version 22.0 (IBM Corp., Armonk, NY, USA) was used for the statistical analyses. The independent *t*-tests were used to analyze the data of age, education, follow-up period, and the scores of CDR-SB, CASI, IADL, NPI, and MoCA. Whereas the analyses of sex, CDR score, and the history of other relevant diseases were performed by the chi-square test. The Cox regression model of the CDR < 1 cohort was adopted to investigate the contribution of CDR-SB levels and CI groups determined using the CDR-SB levels to convert to CDR ≥ 1. HRs were adjusted for sex, age, education, and the history of other relevant diseases. The conversion and reversion rates for different CI levels were summarized and compared. *p* < 0.05 was considered statistically significant for all statistically analyses.

### Ethical consideration

The study was conducted retrospectively, and the data were processed and analyzed anonymously. The institutional review board of Show Chwan Memorial Hospital approved this study, and waived the informed consent requirement (SCMH_IRB No: IRB1081006).

## Results

A total of 1,827 participants were analyzed, including 1,258 (68.9%) non-converters, and 569 (31.1%) converters with the mean follow-up of 2.1 (0.4–5.5) and 1.8 (0.3–5.0) years, respectively.

The comparison of demographic variables between the non-converters and converters groups without adjustment revealed significant differences in age (*p* < 0.001), education (*p* < 0.001), sex (*p* = 0.004), CDR score (*p* < 0.001), CDR-SB score (*p* < 0.001), CASI score (*p* < 0.001), MoCA score (*p* < 0.001), IADL score (*p* < 0.001), NPI score (*p* = 0.008), CVD (*p* = 0.024), dyslipidemia (*p* = 0.001), and congestive heart failure (*p* = 0.044) (Table 1).

**TABLE 1:** Comparison of demographical data between non-converters and converters groups of the participants with clinical dementia rating (CDR) < 1.

	Non-converters mean (SD)	Converters mean (SD)	*P*-value
N	1,258	569	
Age, year	71.2 (9.8)	77.2 (7.6)	<0.001
Sex, female, N (%)	655 (52.1)	337 (59.2)	0.004
Education, year	6.5 (4.7)	4.7 (4.2)	<0.001
Follow-up, year	2.1 (1.1)	1.8 (1.0)	<0.001
CDR, 0/0.5, N	230/1,028	16/553	<0.001
CDR-SB	1.4 (1.2)	2.5 (1.3)	<0.001
CASI	76.0 (13.9)	64.7 (13.6)	<0.001
MoCA	17.5 (6.7)	12.3 (5.4)	<0.001
IADL	7.0 (1.5)	5.8 (1.9)	<0.001
NPI	3.7 (6.0)	4.5 (6.1)	0.008
Cerebrovascular disease, N (%)	170 (13.5)	100 (17.6)	0.024
Parkinsonism, N (%)	173 (13.8)	90 (15.8)	NS
Hypertension, N (%)	512 (40.7)	232 (40.8)	NS
Diabetes, N (%)	241 (19.2)	128 (22.5)	NS
Dyslipidemia, N (%)	283 (22.5)	91 (16.1)	0.001
Carotid artery disease, N (%)	93 (7.4)	52 (9.1)	NS
Arrhythmias, N (%)	63 (5.0)	27 (4.7)	NS
Congestive heart failure, N (%)	45 (3.6)	32 (5.6)	0.044

CDR, clinical dementia rating scale; N, number; SD, standard deviation; NS, non-significance; CDR-SB, sum of boxes of the CDR; CASI, cognitive abilities screening instrument; MoCA, montreal cognitive assessment; IADL, instrumental activities of daily living; NPI, neuropsychiatric inventory.

**FIGURE 2:**
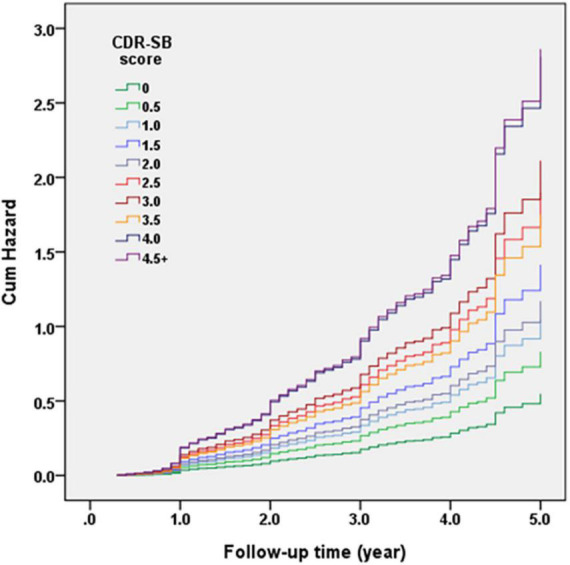
Cox regression model of the clinical dementia rating (CDR) < 1 cohort was adopted for investigating the contribution of sum of boxes of the CDR (CDR-SB) levels to conversion to CDR ≥ 1. Hazard ratios (HRs) were adjusted for age, gender, education, cerebrovascular disease (CVD), parkinsonism, diabetes, hypertension, dyslipidemia, coronary artery disease, arrhythmias, and congestive heart failure.

Figure 2 demonstrates the Cox regression model of the CDR < 1 cohort to investigate the contribution of CDR-SB levels to conversion to CDR ≥ 1. HRs were adjusted for age, sex, education, CVD, parkinsonism, diabetes, hypertension, dyslipidemia, coronary artery disease, arrhythmias, and congestive heart failure. After adjustment, the conversion rate of the group with CDR 0 was the lowest one (7% after a mean follow-up year of 1.7). After excluding the participants with CDR 0, the conversion rate was 35.0% with a mean follow-up year of 2.0. The HR was 3.75 for CDR 0.5 compared to CDR 0. The relationship between CDR-SB and conversion rates showed that conversion rates increased with the increasing CDR-SB levels. Compared to a CDR-SB score of 0, the HRs for conversion to CDR ≥ 1 were 1.51, 1.91, 2.58, 2.13, 3.46, 3.85, 3.19, 5.12, and 5.22 for CDR-SB scores of 0.5, 1, 1.5, 2, 2.5, 3, 3.5, 4, and ≥4.5, respectively. All CDR-SB levels had *p* values of <0.05, except CDR-SB score = 0.5. In addition, older age [HR = 1.04; *p* < 0.001], lower education [HR = 1.03; *p* = 0.009], lower CASI score [HR = 1.02; *p* < 0.001], and a history of diabetes [HR = 1.23; *p* = 0.037] were also associated with increased conversion rates. Based on the results of a significantly different prediction power between a CDR-SB score of 3.5 [HR = 3.19] and 4.0 [HR = 5.12], and a very similar prediction power between a CDR-SB score of 4.0 [HR = 5.12] and ≥4.5 [HR = 5.22], the diagnosis for VMD using the CDR-SB was revised from a CDR-SB score of 2.5–4.0 to that of 2.5–3.5, and that for dementia was revised from a CDR-SB score of ≥4.5 to that of ≥4.0.

**FIGURE 3:**
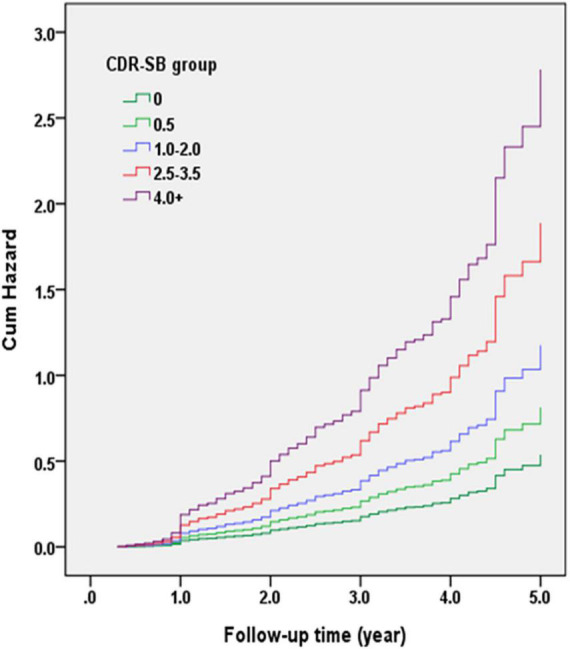
Cox regression model of the clinical dementia rating (CDR) < 1 cohort was adopted for investigating the contribution of cognitive impairment (CI) groups determined with the sum of boxes of the CDR (CDR-SB) levels to conversion to CDR ≥ 1. Hazard ratios (HRs) were adjusted for age, gender, education, cerebrovascular diseases (CVD), parkinsonism, diabetes, hypertension, dyslipidemia, and coronary artery diseases.

Figure 3 demonstrates the Cox regression model of the CDR < 1 cohort to investigate the contribution of the CI groups determined using CDR-SB levels to convert to CDR ≥ 1. The HRs were adjusted for age, sex, education, CVD, parkinsonism, diabetes, hypertension, dyslipidemia, coronary artery disease, arrhythmias, and congestive heart failure. The results showed that the conversion rates increased with the increasing CI stages. Compared to a CDR-SB score of 0 (NC), the HRs for conversion to CDR ≥ 1 were 1.51, 2.18, 3.51, and 5.17 for CDR-SB scores of 0.5, 1.0–2.0 (MCI), 2.5–3.5 (VMD), and ≥4.0 (dementia), respectively. All CDR-SB levels had *p* values <0.05, except a CDR-SB score of 0.5 [*p* = 0.169]. In addition, older age [HR = 1.04; *p* < 0.001], lower education [HR = 1.03; *p* = 0.010], lower CASI scores [HR = 1.02; *p* < 0.001], and a history of diabetes [HR = 1.23; *p* = 0.035] were also associated with increased conversion rates.

**FIGURE 4:**
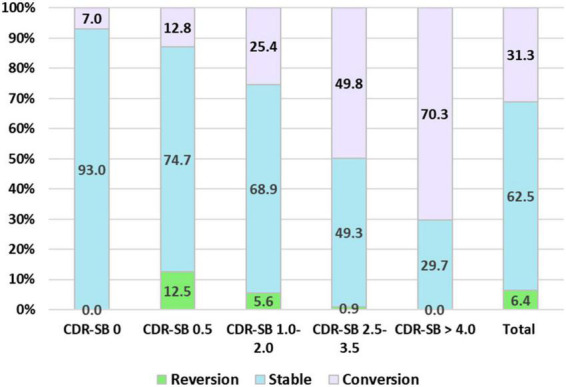
Percentage frequency of reversion to normal cognition (NC), stable, and conversion to clinical dementia rating (CDR) ≥ 1 among different cognitive impairment (CI) groups determined with the sum of boxes of the CDR (CDR-SB) levels.

Finally, the conversion and reversion rates for different CI stages determined using the CDR-SB levels are summarized in Figure 4. After a mean follow-up of 1.7, 2.1, 2.0, 1.8, and 1.6 years, conversion rates to CDR ≥ 1 were 7.0, 12.5, 25.4, 49.8, and 70.3% for the CDR-SB levels of 0, 0.5, 1–2, 2.5–3.5, and ≥4.0, respectively. The annual conversion rates were 4.1, 6.0, 12.7, 27.6, and 43.9% for the CDR-SB levels of 0 (NC), 0.5 (SCD), 1.0–2.0 (MCI), 2.5–3.5 (VMD), and ≥4.0 (dementia), respectively. Paired comparisons were all significant between the different groups. After a mean follow-up of 2.0, 2.1, 2.5, and 1.6 years, the reversion rates were 12.5, 5.6, 0.9, and 0.0% for the CDR-SB levels of 0.5 (SCD), 1.0–2.0 (MCI), 2.5–3.5 (VMD), and ≥4.0 (dementia), respectively. The annual reversion rates were 6.3, 1.3, 0.4, and 0% for the CDR-SB levels of 0.5 (SCD), 1.0–2.0 (MCI), 2.5–3.5 (VMD), and ≥4.0 (dementia), respectively. Paired comparisons were significant between different groups, except a CDR-SB level of 2.5–3.5 (VMD) versus a CDR-SB level of ≥4.0 (dementia).

## Discussion

Recently, researchers have put considerable effort into developing or discovering practical tools for tracing the deterioration of cognition or daily function in the predementia stages. Following this idea, we have investigated the predictive value of the commonly used staging tools for AD, the CDR and CDR-SB, by studying their contribution to conversion or reversion in people without dementia. Several important findings of this study deserve attention. First, the CDR-SB has a high predictive value for detecting conversion to dementia in people without dementia. The conversion rates increased with increasing CDR-SB scores. The conversion rates of CI stages grouped using different CDR-SB levels also had a very good prediction power. The original grouping of participants with CDR 0.5 using the CDR-SB in this study were determined according to the previous studies by O’Bryant et al. (2008, 2010) and Yang et al. (2021). The CDR-SB levels of 0, 0.5, 1.0–2.5, 2.5–4.0, and ≥4.5 were defined as NC, SCD, MCI, VMD, and dementia, respectively. However, during analysis of conversion rates of different CDR-SB levels based on the finding of very similar conversion rates of CDR-SB 4.0 and ≥4.5, we decided to adjust CDR-SB 2.5–3.5 as the diagnosis of VMD and CDR-SB ≥ 4.0 as the diagnosis of dementia in the later analysis. Our results showed that compared to a CDR-SB score of 0 (NC), the HRs for conversion to CDR ≥ 1 were 1.51, 2.18, 3.51, and 5.17 for CDR-SB scores of 0.5, 1.0–2.0 (MCI), 2.5–3.5 (dementia), and ≥4.0 (dementia), respectively. The annual conversion rates were 4.1, 6.0, 12.7, 27.6, and 43.9% for the CDR-SB levels of 0 (NC), 0.5 (SCD), 1–2 (MCI), 2.5–3.5 (VMD), and ≥4.0 (dementia), respectively. These findings suggested that using the CDR-SB for determination of different predementia stages and their conversion rates to dementia are partially consistent with those reported in several previous studies or meta-analyses on the rates of progression from predementia stages to dementia (Petersen et al., 1999; Mitchell and Shiri-Feshki, 2009; Galtier et al., 2019; Slot et al., 2019). For example, SCD, defined using a CDR-SB score of 0.5, showed a tendency to increase the conversion rate to dementia, but not significantly, in this study [HR = 1.51, *p* = 0.169]. The results of most previous studies addressing SCD progression to dementia are controversial and there is no consensus (Mitchell and Shiri-Feshki, 2009; Galtier et al., 2019; Slot et al., 2019). In addition, the annual conversion rate (12.7%) of MCI, defined using a CDR-SB score of 1.0–2.0, was higher than that reported in most previous studies (5–10%) (Petersen et al., 1999; Roehr et al., 2016), indicating that using the CDR-SB in MCI may be able to better predict progression to dementia. In addition, this study included the participants with CDR-SB ≥ 2.5 which indicated one of our study groups was in a stage of VMD. This group (CDR-SB ≥ 2.5) had a highest conversion rate to CDR ≥ 1. Therefore, compared with the previous studies, the conversion rate in our study is relatively high with a mean annual conversion rate > 15% per year.

Second, this study included normal participants and the patients with SCD with CDR 0, SCD, MCI, or VMD with CDR 0.5. According to our results, higher CDR-SB predicted higher conversion rates; therefore, more CDR 0 participants in non-converters than the convertors group is reasonable, and these findings also indicated a good prediction value of CDR-SB for people with non-dementia converting to dementia. In addition, CI stages determined using the CDR-SB levels not only demonstrated high predictive value for conversion to dementia but also predicted reversion to NC (CDR = 0). In this study, after a mean follow-up of 2.1 ± 1.1 years, the reversion rates were 12.5, 5.6, 0.9, and 0.0% for the CDR-SB levels of 0.5, 1–2, 2.5–3.5, and ≥4.0, respectively. Based on the results, the participants with CDR-SB scores ≥ 2.5, considered to indicate VMD and dementia stages in the previous studies, almost did not return to NC; only 0.9% patients with CDR-SB scores of 2.5–3.5 showed reversion to NC, and no participants with CDR-SB scores ≥ 4.0 showed reversion to NC. These findings support that determination of dementia using a CDR-SB score of >2 is logical and reasonable.

Third, in addition to the CDR-SB score, older age [HR = 1.04; *p* < 0.001], lower education [HR = 1.03; *p* = 0.009], lower cognitive performance on the CASI [HR = 1.02; *p* < 0.001], and a history of diabetes [HR = 1.23; *p* = 0.037] were also associated with increased conversion rates after adjustment for important clinical history and vascular risk factors. These findings are consistent with those of previous studies (Galvin et al., 2009; Limongi et al., 2017). However, a history of hypertension [HR = 1.01; *p* = 0.941] or dyslipidemia [HR = 0.92; *p* = 0.479] did not show a significant increase in the incidence of dementia in this study.

Finally, for the determination of conversion or reversion, we used relatively strict criteria. Conversion is defined as deterioration on the clinical assessments, including the CDR, CASI, MoCA, and IADL, without any return to better performance or function in the assessments of the turning point and the following assessments. However, the cohort in this study presented a high conversion rate to dementia and a relatively lower reversion rate (<5%) to NC in the CDR-SB 1.0–2.0 (MCI) stage. The annual conversion rate progression from a CDR-SB score of 1.0–2.0 (MCI) to dementia with CDR ≥ 1 was 12.7%, which is relatively higher than that reported in a previous meta-analysis of 41 studies (Hung et al., 2021). Combining these findings, our study might have provided further evidence that choosing the CDR-SB for the determination of CI stages in people without dementia could be logical and reliable. Tracking predementia stages for conversion or reversion, the CDR-SB might also be a good candidate that can provide a highly predictive value.

There are several limitations to this study that need to be addressed. First, the study was conducted at three centers in Taiwan. Further studies including more centers to investigate the effectiveness of the CDR-SB for tracing conversion or reversion in predementia stages are warranted. Second, not all participants were followed up for equal durations, the follow-up periods were ranged from 0.3 to 5.5 years. Third, this was a retrospective, longitudinal follow-up study, the design was not preplanned, and the data acquired might not be precise or predetermined.

In conclusion, the CDR-SB assessment in predementia stages highly predicts progression to dementia or reversion to NC. Therefore, the CDR-SB has a highly predictive value for tracing cognitive or functional impairment and can be applied to investigate the effectiveness of pharmacological and non-pharmacological interventions.

## Data availability statement

The original contributions presented in this study are included in the article/supplementary material, further inquiries can be directed to the corresponding author.

## Ethics statement

The studies involving human participants were reviewed and approved by the Show Chwan Memorial Hospital. Written informed consent for participation was not required for this study in accordance with the national legislation and the institutional requirements.

## Author contributions

R-CT and Y-WY undertook the literature search and data analysis and were mainly responsible for the revisions and drafts of the manuscript. P-YC participated in the data analysis and contributed to the revisions and final draft of the manuscript. K-CH contributed to the revisions of the manuscript. H-TC undertook the literature search and contributed to revisions. All authors contributed to the article and approved the submitted version.
